# Correlation between Transthoracic Lung Ultrasound Score and HRCT Features in Patients with Interstitial Lung Diseases

**DOI:** 10.3390/jcm8081199

**Published:** 2019-08-11

**Authors:** Milena Adina Man, Elena Dantes, Bianca Domokos Hancu, Cosmina Ioana Bondor, Alina Ruscovan, Adriana Parau, Nicoleta Stefania Motoc, Monica Marc

**Affiliations:** 1 PulmonologyDepartment, University of Medicine and Pharmacy “Iuliu Haţieganu”, 400012 Cluj-Napoca, Romania; 2 Pulmonology Department, Ovidius Medical University, 900470 Constanta, Romania; 3 Pulmonology Hospital “Leon Daniello”, 400371 Cluj Napoca, Romania; 4 Victor Babes Hospital, 300310 Timisoara, Romania

**Keywords:** transthoracic lung ultrasound (LUS), high-resolution computed tomography (HRCT), interstitial lung disease (ILD), B-line, pulmonary interstitial syndrome

## Abstract

Chest high-resolution computed tomography (HRCT) is considered the “gold” standard radiological method in interstitial lung disease (ILD) patients. The objectives of our study were to evaluate the correlation between two transthoracic lung ultrasound (LUS) scores (total number of B-lines score = the total sum of B-lines in 10 predefined scanning sites and total number of positive chest areas score = intercostal spaces with ≥3 B-lines) and the features in HRCT simplified scores, in different interstitial disorders, between LUS scores and symptoms, as well as between LUS scores and pulmonary function impairment. We have evaluated 58 consecutive patients diagnosed with ILD. We demonstrated that there was a good correlation between the total number of B-lines score and the HRCT simplified score (r = 0.784, *p* < 0.001), and also a good correlation between the total number of positive chest areas score and the HRCT score (r = 0.805, *p* < 0.005). The results confirmed the value of using LUS as a diagnostic tool for the assessment of ILD compared to HRCT. The use of LUS in ILD patients can be a useful, cheap, accessible and radiation-free investigation and can play a complementary role in the diagnosis and monitoring of these patients.

## 1. Introduction

Interstitial lung disease (ILD) is a heterogeneous group of disorders with variable etiologies, clinical presentations, radiographic patterns, and histological appearances that affects the lung parenchyma (the interstitium, airspaces, peripheral airways, and vessels) and is challenging to diagnose [1,2]. Classification of idiopathic interstitial pneumonias (IIPs) defined in 2002 based on clinical, radiological and pathological criteria, was updated in 2013 by American Thoracic Society (ATS)/European Respiratory Society (ERS) [3]. ILDs could have a known origin (environmental or occupational exposures, drugs, infections) or could be idiopathic with possible genetic susceptibility of the host (idiopathic interstitial pneumonias, sarcoidosis, respiratory bronchiolitis-associated interstitial lung disease), and early diagnosis can impact the patient’s prognosis [4,5,6,7]. The pathogenesis of fibrosis is complex and can be specific for different agents with disruption of the normal lung architecture [8]. Because it is not sensible to use an X-ray for ILD detection (which can be normal in most patients with early disease), high-resolution computed tomography (HRCT) is considered the “gold” imaging standard method for diagnosis and monitoring of ILD. However, numerous studies have validated the utility of different lung ultrasound (LUS) scores and correlation with computed tomography (CT) scan findings in acute and chronic disease [9,10,11,12]. The clinical presentation, history of antigen/drug or occupational exposure, HRCT images and sometimes characteristic histopathological findings can be highly suggestive for specific diagnoses in patients with ILD. Current data has shown that LUS, a non-invasive technique, may be useful to detect ILD by evaluating B-lines, the sonographic marker of pulmonary interstitial syndrome [6]. Because air is not a favorable medium for transmission of waves, the lung parenchyma has always been considered a “forbidden zone” for ultrasound. LUSs were originally limited to the assessment of superficial pleural conditions, such as tumors, condensations, and effusions, and to guide invasive procedures [13]. In a normally aerated lung, the only detectable structure is the pleura (visualized as a hyper-echoic horizontal line, A-lines, thin and not larger than 2 mm). In the last decade, LUSs have been extensively explored, and they have shown to be highly sensitive to variations in the pulmonary content and the balance between air and fluids, which correlates with lung weight and density by CT scan [14]. According to the International Consensus Conference on Pleural and Lung Ultrasound, B-lines (generated by the reflection of ultrasound beams from thickened interlobar septa) are discrete, laser-like, vertical, hyper-echoic lines that appear from the pleural line and obliterate the majority of A-lines, and move synchronously with lung sliding [15,16,17].

Interstitial syndrome is defined by ≥3 B lines between two ribs in a single scan, which arise from the pleural line, are visible in a frozen image of a longitudinal scan, and display a distance no more than 7 mm between two lines [9,18]. Zawada confirmed examination by keeping the probe perpendicular to the ribs in the longitudinal plane [17]. B-line artifacts can form patterns corresponding to interstitial syndromes, alveolar-interstitial syndromes, or white lungs [19].

Interstitial syndrome that displays thickening of interlobular septa or has a ”ground glass” appearance on radiological imaging implies increased extra vascular lung water content or density in either, or both, the interstitium and alveolar air spaces. The degree of aeration loss, distribution of parenchymal involvement (focal or diffuse), and signs with B-lines differ between diseases and make it possible to differentiate them from LUS. It has been demonstrated that a distance of 7 mm between the B-lines at the pleural line is caused by moderate decrease in lung aeration due to thickened interlobular septa, and it is characteristic of interstitial edema. B-lines that are ≤3mm apart are more characteristic for alveolar edema because this type of edema is caused by a severe decrease in lung aeration, due to fluid (transudate)-filled alveoli which corresponds to ground-glass opacities on CT scan [17]. Anile et al., using LUS, demonstrated good correlation between the number of lung areas positive for B-lines and extravascular lung water [20]. Many studies have assessed the utility of LUS as a semi-quantitative score to measure the lung aeration loss caused by different lung pathologic features in patients with shock, acute respiratory failure, overhydration from nephrology, cardiologic pathologies, and patients that are critically ill [11,17,21,22,23,24,25,26,27,28]. For chronic diseases, studies have demonstrated the utility of LUS as a screening tool to evaluate ILD in connective tissue diseases (CTDs) [10,14,18,29,30,31,32].

The objective of our study was to evaluate the correlation between the LUS score (total number of B-lines score and total number of positive chest areas score, both ultrasound scores) and the features in HRCT simplified scores. The secondary objective was to assess LUS correlation with the presence of symptoms (evaluated by Borg tests with dyspnea and fatigue scores, 6 min walking tests, and significant desaturation) and with the pulmonary function impairment in patients with ILD.

## 2. Materials and Methods

### 2.1. Study Population

Between September 2017 and September 2018, we evaluated in this observational study, 58 consecutive patients diagnosed with interstitial lung disease (age >18 years), who were hospitalized in our Pulmonology Clinic. The study was conducted in accordance with the principles of the Declaration of Helsinki.

### 2.2. Study Protocol

The study was approved by the Ethics Committee of Leon Daniello Cluj Napoca Pulmonology Hospital (2807/28.06.2017). Diagnoses were made according to the respective international criteria based on clinical presentation, HRCT findings, serological tests, pulmonary function tests (PFTs), fiberbronchoscopic findings and bronchoalveolar lavage. All patients underwent procedures after they signed written informed consent forms (see Supplementary Materials).

### 2.3. Clinical Assessment

All patients were clinically evaluated; answered a questionnaire about personal history, environmental and drug exposure, and symptoms (Borg test dyspnea score and Borg test for fatigue pre- and post-effort); and performed a 6 min walking test to assess distance versus significant desaturation (more than 3%).

### 2.4. HRCT Assessment and Disease Quantification

HRCT examinations were performed by a radiologist using a Siemens Somaton Scope CT ((manufacturer Siemens Healthcare Gmbh (Berlin, Germany, software Syngovia) following standard protocol. A Philips medical system was used, and a PPPPP helical CT scanner (Siemens, Forchheim, Germany) was used, without contrast agents, to measure full inspiration from apex to the lung base in patients in the supine position. Parameters were sequentially acquired at 1mm collimation, 10 mm intervals, 220 mA average tube current, and 120 KV tube voltage. Volumetric scans were performed at a high spatial resolution (1 mm thickness) and interval image reconstruction rate during deep inspiration in the supine position. Parenchymal abnormalities on HRCT were coded and scored in all images, and radiologists were blinded to the patient’s clinical and hemodynamic information. For clinical reasons, we adjusted Warrick’s tomography scale to a semi-quantitative score to quantify fibrosis. This scoring system is based on that reported by Wangkaew et al. [29]. We used HRCT to categorize patterns of the lung parenchyma findings representing ILD. Parenchymal abnormalities were classified into four categories: lung fibrosis (Fib) = thickening of interlobular septae or intralobular septae; traction bronchiectasis due to fibrosis (B) = dilatation of bronchial tree with peribronchial wall thickening; ground-glass opacity (GG) = hazy parenchymal opacity with preserved underlying bronchovascular structure without architectural distortion, and honeycombing (HC) = clustered air-filled cyst with dense walls. The extent of pulmonary parenchymal abnormality was scored from each lobe of right and left lung, using a Likert scale (0 = absent; 1 = 1–25%; 2 = 26–50%; 3 = 51–75%; 4 = 76–100%). The total CT fibrosis scores (t-Fib), total CT bronchiectasis scores (t-B), total CT ground-glass opacity (t-GG), total CT honeycombing scores (t-HC) and a total CT score were calculated by summing all scores from all five lung lobes. The diagnostic algorithm of idiopathic pulmonary fibrosis (IPF) requires the presence of a usual interstitial pneumonia (UIP) pattern on HRCT with the presence of honeycombing and reticular abnormalities with a predominantly basal and subpleural distribution. Absence of honeycombing is recognized as a possible/probable UIP pattern. For clinical reasons, the UIP and possible/probable UIP was highlighted in UIP patterns and represents group 1. Nonspecific interstitial pneumonia (NSIP) patterns were defined as peripheral, subpleural, and basal lungs with subpleural sparing and with extensive ground-glass opacity. According to HRCT findings, the patients were divided into three groups: group 1 with UIP/UIP patterns, group 2 with NSIP/NSIP patterns, and group 3 with micronodular patterns or other type of radiological interstitial abnormalities that did not fall under the new international recommendations.

### 2.5. LUS Assessment and Quantification

**Figure 1:**
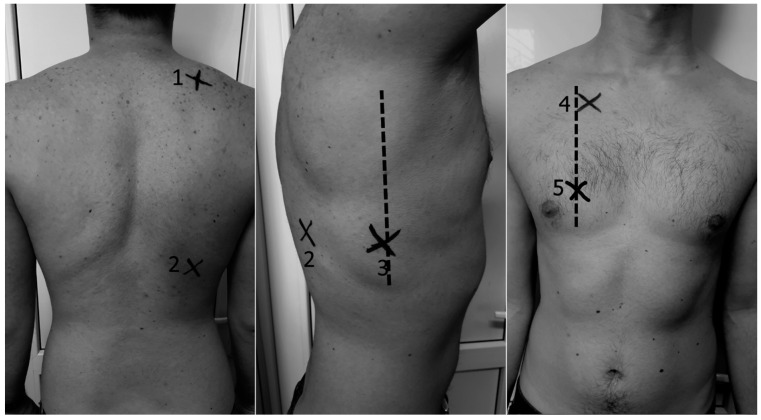
Lung intercostal spaces assessment in a reduced lung ultrasound (LUS) protocol (10 lung intercostal spaces). (**1**) Posterior suprascapular intercostal space (IC); (**2**) posterior basal subscapular IC (space 5–7); (**3**) lateral basal 5–7 IC along midaxillary line; (**4**) anterior 2 IC along midclavicular line; (**5**) anterior basal 5–7 IC along midclavicular line.

After diagnostic exam and HRCT, all patients underwent LUS in 10 intercostal spaces: two posterior (suprascapular bilateral, subscapular in basal spaces 5–7), one lateral (axillary midline basal spaces 5–7), and two anterior spaces (space 2 intercostal anterior along midclavicular line, basal spaces 5–7 along the midclavicular line) for a total 5 chest areas per side (Figure 1). LUS was performed by a pulmonologist trained in chest sonography (blinded to the patient’s clinical and hemodynamic information) using an ultrasound scanner (model Edan, manufacturer: Shanghai International Holding Corp Gmbh, Europe, Hamburg, Germany, C3431) equipped with a 3.5 MHz convex probe in transversal scan (i.e., aligned with the intercostal space) [33].

Assessments of B-lines features (distribution and pattern) were made. The number of B-lines was recorded and summed. The total sum of B-lines visualized in all the explored areas represents the total number of B-lines score. The simplified score was computed as the sum of positive chest areas, defined by the presence of ≥3 B lines, thus ranging from 0 to 10 in the predefined scanning sites (total number of positive chest areas score) [6,10,16]. We used these both of these scores and checked the correlations with HRCT score.

### 2.6. Pulmonary Function Tests (PFTs)

Standard spirometry was performed in all patients by Viasys Master Screen Spirometer Body/DFF (manufacturer Viasys Healthcare GmbH, Höchberg, Germany), and the following parameters were measured: forced expiratory volume (FEV), forced vital capacity (FVC), and FEV/FVC expressed in actual value and as a percentage of predicted values. More than 80% was considered normal, 60–79% was considered mild dysfunction, 40–59% moderate dysfunction, and less than 39% was considered severe dysfunction. The DLCO (diffusing capacity of the lung for carbone monoxide) was determined as the single-breath diffusing lung capacity and corrected for hemoglobin and carbon monoxide (CO) levels, and the results were registered as percentages of predicted values.

An arterial blood gas test (ABG) was performed using an Opti CCA-TS2 blood gas analyzer. Blood samples were drawn from patients in ambient air through puncture of the radial artery, and then the values of arterial partial oxygen pressure (PaO2) and arterial partial carbon dioxide pressure (PaCO2) were recorded.

### 2.7. Statistical Analysis

For statistical analysis we used SPSS software version 25.0. Results are expressed as mean ± standard deviation, or median (25th–75th percentile), depending on the number and percentage. Univariate comparisons were made with χ^2^, 2-sample *t* tests, and Mann–Whitney test. If we had to compare three groups, we used an Anova test or Kruskall–Wallis test followed by Scheffe post hoc analysis. We applied Pearson’s correlation coefficient in the case of two quantitative, normally distributed variables. We applied Spearman’s correlation coefficient between quantitative variables not normally distributed or categorical variables. The sample size for correlation was generated using Power Analysis and Sample Size Software (PASS) version 11.0 (NCSS statistical software, Keysville, UT, USA) with *p*-value = 0.05 and power = 80% [34]. We consider a value between 0.6 to 0.8 as an objective to use Pearson’s correlation coefficient [35].

## 3. Results

This study was carried out with 58 patients consecutively diagnosed with ILD, 34 men (58.6%) and 24 women (41.4%), with a mean age of 58.97 ± 15.59 years.

In our study group, we found an HRCT score of 21.66 ± 7.79 and a total number of B-lines score of 78.72 ± 44.31. The total number of positive chest areas score was 7.5 (range 4–10). Maximum total number of B-lines score was 174, and the minimum 0; the maximum HCRT score was 40, and the minimum was 5; and the maximum total number of positive chest areas score was 10, and the minimum was 0.

**Figure 2:**
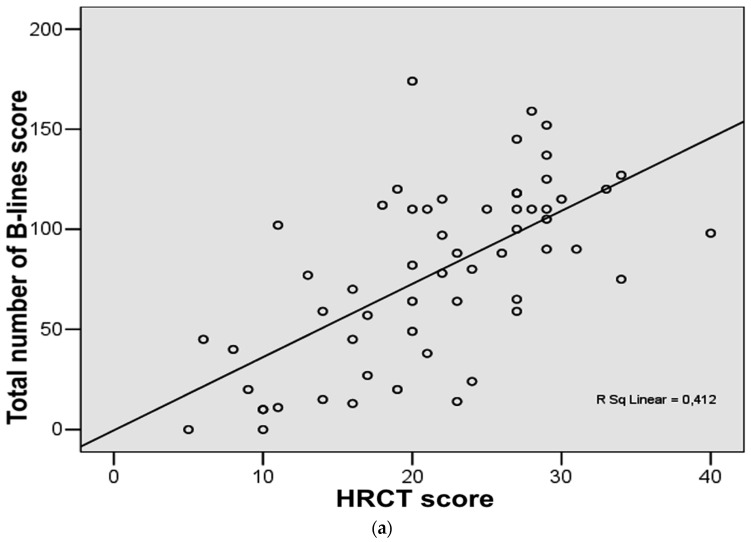

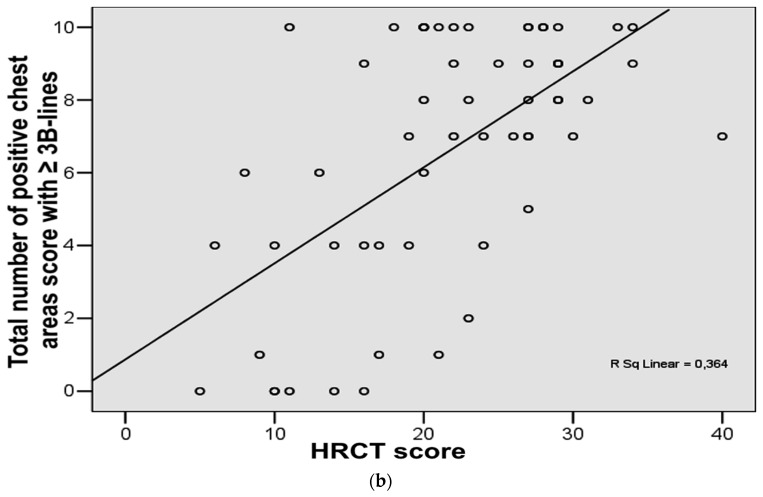
(**a**) Correlation between chest high-resolution computed tomography (HRCT) score and total number of B-lines score in the study group. (**b**) Correlation between chest high-resolution computed tomography (HRCT) score and total number of positive chest areas score with ≥3 B-lines in the study group.

The analysis of the degree of relationship in ILD patients (*n* = 58) showed a good correlation between the total number of B-lines score and HRCT score (r = 0.64, *p* < 0.001) (Figure 2a). Good correlation was found between total number of positive chest areas score (with ≥3 B-lines) and HRCT score (r = 0.60, *p* < 0.001) (Figure 2b).

From the 58 patients consecutively diagnosed with ILD, 30 (51.7%) were diagnosed with IPF, 7 (12.1%) with NSIP, 9 (15.5%), with pulmonary sarcoidosis, and 12 (20.7%) patients were diagnosed with CTD including 5 (8.62%) patients with rheumathoid arthritis, 5 (8.62%) patients with systemic sclerosis (SSc), 1 (1.72%) patient with systemic lupus erythematosus, and 1 (1.72%) with ankylosing spondylitis. Age was significantly different between the three groups (*p* < 0.001) (Table 1). In the first group (UIP pattern), the patients were significantly older than the others.

The HRCT score was calculated with the most significant lesions for the UIP group. A significant difference was found between the UIP (group one) and NSIP (group two) groups compared to group three (micronodules or other radiological interstitial abnormalities) (Table 1).

The mean values for the total number of B-lines score were determined for the entire group, and the highest values were found for the UIP group. There was a significant difference between groups, *p* < 0.001 (Table 1). The total number of positive chest areas score was significantly different between the three groups *p* < 0.001 (Table 1).

**Figure 3:**
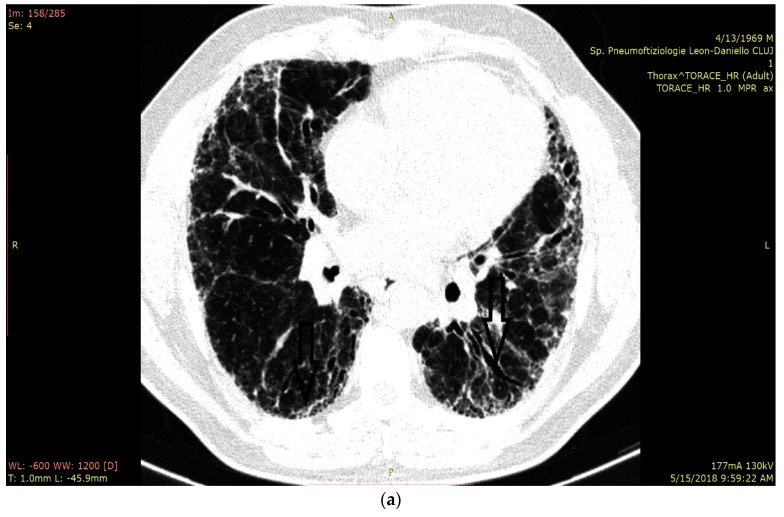

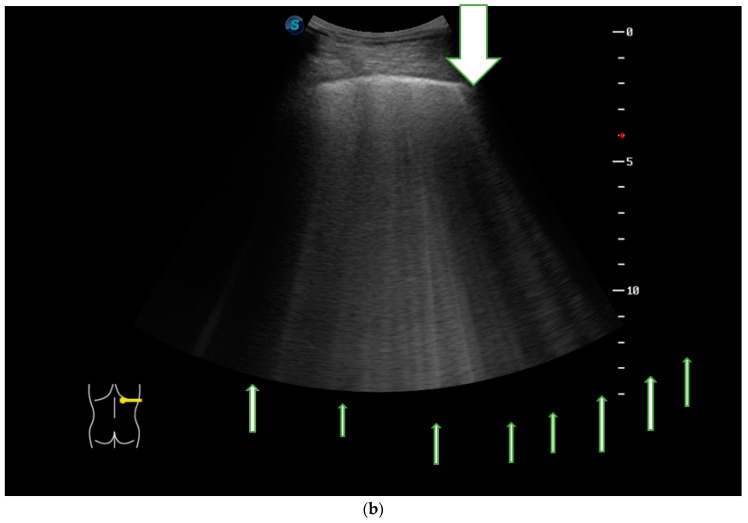
(**a**) HRCT imaging in the lung window at the level of middle and lower lobes with subpleural honey combing and fine reticulation, as well as traction bronchiectasis, that confirm UIP pattern (black lines). (**b**) LUS imaging with multiple B-lines (thin arrow), confirm the fibrotic interstitial syndrome; thick arrow represents pleural line which is not thickened.

In Figure 3a we present the HRCT changes in the UIP pattern, and in Figure 3b we highlight the presence of B-lines in LUS (Figure 3a,b).

SaO2 at rest was measured by pulsoximetry for all the patients. From the total number of patients included in the study (*n* = 58), 86.7% had an SaO2 under 90% and 13.3% over 90%. We found a significant difference between the UIP group and the NSIP group compared to patients in group three (with micronodules or other radiological interstitial abnormalities) (*p* = 0.010, Table 1). PaO2 values were assessed for all the patients, and hypoxemia at rest was not found to be statistically different between groups (*p* = 0.707).

A statistical difference between groups was also found for the DLCO values. No significant difference was described between the groups for the Borg dyspnea scale, pre- or post-effort (*p* = 0.488), and Borg fatigue scale pre- or post-effort (*p* = 0.066), nor for the pulmonary hypertension values (see Table 1).

The total number of B-lines score had a positive correlation with age (r = 0.481, *p* < 0.001), a negative correlation with DLCO (r = −0.39, *p* = 0.007), and a negative correlation with VA (r = −0.39, *p* = 0.007).

The total number of positive chest areas score had a positive correlation with age (r = 0.599, *p* < 0.001) but a negative correlation with DLCO (r = −0.44, *p* = 0.002) and VA (r = −0.49, *p* = 0.001).

HRCT scores had a positive correlation with age (r = 0.343, *p* = 0.008) and negative correlations with SaO2 (r = −0.343, *p* = 0.008), PaO2 (r = −0.387, *p* = 0.020), FVC (r = −0.277, *p* = 0.037), DLCO (r = −0.592, *p* < 0.001) and VA (r = −0.625, *p* < 0.001).

The correlations between LUS scores or HRCT score and clinical signs were weak or moderate. 

## 4. Discussions

In this study, the main objective was to evaluate the correlation between two LUS scores (total number of B-lines score—the total sum of B-lines in ten predefined scanning sites—and the total number of positive chest areas score, with ≥3 B-lines) and HRCT features in ILD patients. We demonstrated a good correlation between the total number of B-lines score and the HRCT simplified score and also a good correlation between the total number of positive chest areas score and HRCT score. These results sustained the value of using LUS as a diagnostic tool for the assessment of ILD when compared to HRCT, the “gold” standard diagnostic. Because there are not many studies for UIP, splitting the patients into three distinct groups (UIP, NSIP and other radiological abnormalities) could reveal significant difference in ultrasound evaluation in patients with ILDs. The pulmonary lesions in UIP pattern (honey combing, reticular opacities, traction bronchiectasis) are typically distributed subpleural with a basal predominance and these lesions (pattern UIP) may be more easily visible on LUS. NSIP pattern was defined as peripheral, subpleural, and basal lungs with subpleural sparing, with extensively ground glass opacity and may be less visible on LUS. For other ILDs (like sarcoidosis) the utility of LUS may be less significant because there are micronodular lesions in the middle lung distribution.

In the first study about B-lines, in 2009, in patients with SSc with ILDs, a B-line score was calculated on 72 scanning sites by summing the total number of B-lines, and the examination was considered positive when the B-line sum in all scanning sites was >10 [10]. Gutierrez et al. compared two different LUS methods to assess the number of B-lines in 50 scanning sites and in 14 scanning sites and found a significant correlation between the two scoring systems (*p* = 0.0001) [30]. Recently, fewer scanning sites (only 10 sites) in patients with CTD-associated ILD have been evaluated, and it has been found that this modified and simplified scoring system had a good correlation with HRCT (correlation coefficient = 0.695, *p* < 0.001) and was less time-consuming (mean 5.4 ± 1.8 min) [31]. For patients with acute respiratory distress syndrome or for patients from Intensive Care Units, some of authors used a 12-region scan and described four ultrasound aeration patterns: normal aeration (N)—lung sliding with A-lines, less than 3 B-lines; moderate loss of lung aeration (B1 lines)—a clear number of visible B-lines with horizontal spacing between adjacent B-lines ≤7 mm; severe loss of lung aeration (B2 lines)—multiple B-lines fused together, difficult to count with horizontal spacing between adjacent B-lines ≤3 mm, including “white lung”; and consolidation (C)—hyperechoic lung tissue [21,22,24,25,26].

In a recent study (on 34 CTD patients), Tardella et al. reported that the presence of more than 10 B-lines on lung sonography (representative for “lung interstitial syndrome”) can be used as a cutoff point for a high probability of ILD in SSc patients [1,32]. Asano et al. reported good correlations between B-lines and the extent of the reticular pattern on HRCT (r = 0.710; *p* < 0.01) [36].

We used Asano et al.’s study for our sample size because the study was performed on 40 patients (16 with IPF and 24 with NSIP), with 12 locations overall, and the results showed good correlations between lung comet-tail numbers and the extent of reticular patterns on HRCT (r = 0.710; *p* < 0.01), predicted FVC (r = −0.614; *p* < 0.01), and DLCO (r = −0.577; *p* < 0.01). The lung comet-tail number had a strong, negative correlation with the percutaneous SaO2 level after the 6 min walk test (r = −0.751; *p* < 0.01). We considered a value between 0.6 to 0.8 as an objective to use Pearson’s coefficient of correlation because the diseases were diverse (UIP pattern, NSIP pattern, and other findings on HRCT). Some of these diseases show lesions with more central localization, not subpleural, where LUS is not useful [36].

In the last decade, LUS evaluation has been extensively explored for CTD-associated ILD for acute conditions such as cardiogenic pulmonary edema (CPE) and noncardiogenic alveolar interstitial syndrome, viral pneumonia, or pulmonary hypertension [37,38].

We know that the main symptoms correlate well with HRCT, PFT, and the 6 min walking test. We found a correlation between the total number of B-lines score and total number of positive chest areas score using certain parameters, like Borg test dyspnea, and desaturation after 6 min walking test, with differences between the three groups.

Cakir et al. also reported B-lines negatively correlated with DLCO (r = −0.56; *p* = 0.0001) and FVC (r = 0.46; *p* = 0.001) [39]. In another study, Tardella et al., after evaluating 14 lung intercostal spaces in patients with SSc, detected that a cutoff = 10 B-lines was predictive for HRCT presence in significant SSc-ILD patients, and they confirmed correlations with HRCT (rho = 0.819; *p* < 0.001), DLCO (rho = 0.600; *p* < 0.001), and with health-related quality of life variables (rho = 0.560; *p* < 0.001) [32]. B-lines artifact are reproducible, can be easily identified and are easy to learn by operators with different skill and expertise (Dietrich reported a 0.94 Kappa statistic value in 1200 examinations) [40]. LUS is an attractive technique, which can become an important clinical tool to be integrated with HRCT, PFT and six minute walk tests in the screening and evaluation of ILD. B-lines are waiting to be fully validated in CTD, and new data are still to be released for all ILD [13,41,42]. In resource-limited settings, LUS could be considered a particularly useful diagnostic modality in the evaluation of interstitial syndromes.

There is no consensus on specific ultrasound diagnostic criteria in defining ILD (most studies included a small cohort of patients and calculated a total B-line score, a semi-quantitative score, defining ILD as >5 or >10 B-lines, or the distance between B-lines). New multicenter studies are needed to demonstrate validity, reliability, and responsiveness. New diagnostic criteria and standardized techniques must be elaborated by experts before eventually including LUS in the algorithm of diagnosis for symptomatic patients before being sent to HRCT [37]. Point-of-care ultrasound (POCUS) is described as the stethoscope of the future and is being implemented across the medical field by clinicians for bedside examination of patients [43]. Patients with symptoms and positive LUS scores must be sent to HRCT because ultrasound explores only the pleural and subpleural areas of the lung, does not provide information of the deeper zones and parenchymal details, does not correlate with lung histology, and does not predict the prognosis of the disease [9].

The limitations of our study are represented by the small number of patients (especially for the second group with NSIP and third group with other interstitial disease like sarcoidosis) and small number of patients with early interstitial disorders. The later can be explained by the fact that these patients seek treatment at advanced stages of disease or are later directed to specialized centers, as there is no easy accessible method of screening for ILD.

Using a semi-quantitative HRCT score (for clinical reasons to shorten the time to evaluate patients) is also a limitation. LUS is a time-consuming procedure, but using only 10 sites can improve the examination time to only a few minutes. Also, LUS can be useful for initial diagnosis of interstitial syndromes in a dyspneic patient with acute or chronic symptoms. It is not useful in ILD without subpleural lesions (e.g., pulmonary sarcoidosis with perihilar lesions), as it cannot differentiate the etiology of interstitial syndrome, but is useful in monitoring possible pulmonary complications like pleural effusion or pneumothorax.

Another limitation would be the use of an observational study; thus, we cannot evaluate a negative predictive value (NPV) for this method nor a positive predictive value (PPV) because our study included a hospitalized population, not the general population.

We consider the results of this study relevant because there was a high percentage of IPF patients (30 of out 58 patients), two LUS scores were used (the total number of B-lines score in 10 predefined scanning sites, which is a simplified score that explored both upper and lower lobes, and the total number of positive chest areas score), and there was concordance between the scores, respectively, that correlated with the simplified HRCT score (semi-quantitative score, which was not perfect, but was less time consuming).

These clinical methods can be applied in practice and are suitable for a quick assessment of symptomatic dyspneic patients, which can eventually be used for screening the risk in patients and selecting patients for HRCT.

## 5. Conclusions

The use of LUS in ILD patients can be a useful, cheap, accessible, and radiation-free investigation. Our study has shown that LUS (both ultrasound scores: total number of B-lines score and total number of positive chest areas score) can play a complementary role in the diagnosis and monitoring of ILD patients. We also found a good correlation between HRCT and both ultrasound scores, but more studies and standardized methods are needed.

## Figures and Tables

**Table 1:** Comparison between group one (usual interstitial pneumonia, UIP), group two (nonspecific interstitial pneumonia, NSIP), and group three (micronodules or other radiological interstitial abnormalities).

Parameters	Total (*n* = 58)	UIP (*n* = 35)	NSIP (*n* = 11)	Micronodules or Other Radiological Interstitial Abnormality (*n* = 12)	*p*
Male gender no. (%)	34 (58.6)	23 (65.7)	5 (45.5)	6 (50.0)	0.391
Age (years)	58.97 ± 15.59	66.03 ± 11.37 ^a,b^	53.55 ± 11.37	43.33 ± 16.95	<0.001
Diagnosis of CTDs (connective tissue diseases) no. (%)	10 (17.2)	5 (14.3)	3 (27.3)	2 (16.7)	0.609
Oxygen saturation (SaO2) %	95 (93–96)	94 (91–96)^b^	94 (93.5–95.5) ^c^	97 (95.5–97)	0.010
PaO2 (at rest) mmHg	65.74 ± 13.21	64.6 ± 13.52	68.18 ± 10.44	69.53 ± 17.03	0.707
DLCO (diffusing capacity of the lung for carbone monoxide) (%Predicted value)	44.8 (31.45–53.05)	40.2 (30.65–48.6) ^b^	42.8 (33.35–53.05) ^c^	67.7 (49.65–80.35)	0.003
Alveolar volume (VA) (%)	62.59 ± 18.60	60.00 ± 16.72 ^b^	54.23 ± 18.64	78.46 ± 20.21	0.035
HRCT(chest high-resolution computed tomography) score	21.66 ± 7.79	23.94 ± 6.62 ^b^	22.64 ± 9.05 ^c^	14.08 ± 4.91	<0.001
Total number of B-lines score (total sum of B-lines)	78.72 ± 44.31	101.77 ± 32.94 ^a,b^	63.91 ± 36.46 ^c^	25.08 ± 23.75	<0.001
Total number of positive chest areas score (with ≥ 3 B-lines)	7.5 (4–10)	9 (8–10) ^a,b^	7 (4–7) ^c^	0.5 (0–4)	<0.001
Borg fatigue scale	2 (0.5–3)	2 (1–3)	1 (0–2)	1 (0–2.5)	0.066
Borg dyspnea scale	0 (0–2)	0.5 (0–2)	0 (0–1)	0 (0–1)	0.488
Pulmonary hypertension on echocardiographic exam mmHg	34.98 ± 18.56	37.75 ± 19.19	32.75 ± 19.62	23.00 ± 6.71	0.249
FVC (forced vital capacity) (L)	2.63 ± 0.93	2.48 ± 0.69 ^b^	2.45 ± 1.01	3.31 ± 1.27	0.024
FVC (%)	76.91 ± 23.47	77.35 ± 22.07	65.20 ± 24.10	87.22 ± 24.06	0.086

Mean ± standard deviation for normal distributed variables; median (25th–75th percentile) for non-normal distributed variables; no. (%); ^a^—*p* < 0.05 when UIP were compared with NSIP, ^b^—*p* < 0.05 when UIP were compared with group three (micronodules or other radiological interstitial abnormalities); ^c^—*p* < 0.05 when NSIP were compared with group three.

## Supplementary Materials

The following are available online at https://www.mdpi.com/2077-0383/8/8/1199/s1.
